# Natural Antioxidants and Aquatic Animal Health

**DOI:** 10.3390/antiox14020185

**Published:** 2025-02-05

**Authors:** Rui Jia

**Affiliations:** Key Laboratory of Integrated Rice-Fish Farming Ecology, Ministry of Agriculture and Rural Affairs, Freshwater Fisheries Research Center, Chinese Academy of Fishery Sciences, Wuxi 214081, China; jiar@ffrc.cn

## 1. Introduction

In intensive aquaculture, aquatic animals inevitably encounter multiple stressors due to environmental fluctuations, triggering various stress responses, with oxidative stress being the most prevalent [1,2]. Numerous factors within the aquatic environment, including stocking density, temperature, salinity, heavy metals, bacteria, and viruses, can induce the production of reactive oxygen species (ROS), leading to oxidative stress [3,4,5]. Excessive ROS can trigger lipid peroxidation, protein misfolding, and DNA damage in aquatic animals, leading to tissue damage and impaired physiological functions [6]. Prolonged oxidative stress can decrease growth performance, compromise the immune system, and potentially lead to mortality [7]. Furthermore, the development and progression of various diseases in aquatic animals have been conclusively linked to oxidative stress [8]. To mitigate the adverse effects induced by stressors, promising research results have shown that incorporating medicinal plants and their extracts into diets is an effective and eco-friendly strategy [9]. Many medicinal plants, rich in active compounds such as polysaccharides, alkaloids, tannins, saponins, glycosides, flavonoids, steroids, and essential oils, have shown significant antioxidant properties [10]. However, the relationship between antioxidants and the health of aquatic animals still necessitates extensive research, as the primary molecular mechanisms and pathways underlying these beneficial effects remain largely unclear.

The Special Issue “Natural Antioxidants and Aquatic Animal Health” is designed to elucidate the critical role of natural antioxidants in enhancing the health and well-being of aquatic species. It provides insights into the mechanisms through which aquatic animals respond to oxidative stress and how dietary antioxidants impact their health. Here, we present an overview of the Special Issue, which encompasses 12 original articles.

## 2. Overview of Published Articles

Antioxidants, used as dietary additives in aquatic animals, have attracted considerable attention due to their multifaceted roles, such as promoting growth, enhancing antioxidant status, and improving metabolic functions [11]. Jia et al. [Contribution 1] discovered that dietary proanthocyanidins, a natural antioxidant, enhanced muscle nutrients by increasing antioxidant capacity and the levels of polyunsaturated fatty acids (n-3 and n-6) in *Cyprinus carpio.* Furthermore, their research revealed that proanthocyanidins modified intestinal functions through the upregulation of the sphingolipid catabolic process and the lysosomal pathway. This was coupled with a reduction in intestinal cholesterol absorption and an increase in the diversity of the intestinal microbiota. Interestingly, Cheng et al. [Contribution 2] documented that microbial antioxidants, novel compounds fermented by probiotics, enhanced growth, boosted levels of antioxidant enzymes (T-SOD and Gpx), and, as a result, improved survival rates of *Procambarus clarkii*. The work from Song et al. [Contribution 3] focused on the effects dietary lysophosphatidylcholine (LPC) on uptake of astaxanthin, an efficient antioxidant in shrimp feeds. The results showed that LPC supplementation could facilitate the deposition of dietary astaxanthin into farmed shrimp, thereby increasing its beneficial effects. Also, LPC could independently influence the regulation of body color and cholesterol transport in *Litopenaeus vannamei*.

It is noteworthy that various studies have focused on the use of antioxidants to alleviate adverse stimuli and improve health in aquatic animals. In the Gibel carp (*Carassius auratus gibelio*) [Contribution 4], two prevalent antioxidants, taurine and vitamin C, found extensively in various medicinal plants, have been shown to improve growth, antioxidant capacity, immunity, and hypoxia tolerance. Similarly, berberine, a natural alkaloid prevalent in various medicinal plants and known for its antioxidative properties, has shown protective effects in *Oreochromis niloticus* [Contribution 5]. Here, berberine protected against liver damage induced by a high-fat diet (HFD) through the regulation of lipid metabolism, enhancement of antioxidant status, and modulation of immune responses, likely mediated by the Nrf2, TLR2/MyD88/NF-κB, and PPARα signaling pathways. An in vitro study demonstrated that NAD+ precursors mitigate hepatocyte damage caused by high glucose levels in *Megalobrama amblycephala* [Contribution 6]. This protective effect is facilitated through the activation of Sirt1/3, enhancement of redox defense, inhibition of inflammatory responses and apoptosis, and modulation of glucose metabolism. Jiang et al. [Contribution 7] found that dietary supplementation with phosphatidylserine (PS) mitigated stress responses, redox imbalances, and immunosuppression induced by high stocking density in *M. amblycephala*. They noted that the Nrf2 pathway plays a critical role in the beneficial effects of PS.

It has been reported that the antioxidant defense system plays a crucial role in how aquatic animals respond to adverse stimuli [12]. Jin et al. [Contribution 8] found that alkalinity exposure led to a rising trend in superoxide dismutase (SOD) activity and also impacted the oxidative phosphorylation signaling pathway in *Macrobrachium nipponense*. The study by Xue et al. [Contribution 9] indicated that long-term high-fat diet feeding in *Aplodinotus grunniens* induced oxidative stress, which suppressed antioxidant capacity and increased both apoptosis and autophagy in gut cells. He et al. [Contribution 10] evaluated the response mechanisms of *Eriocheir sinensis* to oxidative stress triggered by hydrogen peroxide (H_2_O_2_), a prevalent oxidant. Their findings revealed that low concentrations of H_2_O_2_ activated the antioxidant system, enhancing the organism’s ability to cope with adverse stimuli. Conversely, high concentrations of H_2_O_2_ decreased the antioxidant capacity, suggesting that while mild oxidative stress activates the antioxidant system as a defense mechanism, excessive oxidative stress overwhelms this system and accelerates cellular damage. A study from Liang et al. [Contribution 11] suggested that inadequate nutrition has been linked to decreased levels of key antioxidant enzymes—superoxide SOD, catalase (CAT), and glutathione peroxidase (GPx)—in *Micropterus salmoides*, potentially due to the inhibition of the Nrf2 pathway. Additionally, antioxidative parameters were employed to assess and optimize anesthetic practices for mud crabs (*Scylla paramamosain*), and it was found that clove oil is a safe and optimal anesthetic agent for this species, as it exerts no physiological stress [Contribution 12].

## 3. Conclusions

These research articles in this Special Issue highlight the beneficial effects of antioxidants as dietary additives and provide a theoretical basis for their application in aquaculture. Additionally, these studies offer new insights into the role of antioxidant defense systems in mitigating adverse stimuli in aquatic animals. However, the relationship between natural antioxidants and aquatic animals is rendered complex due to the diversity of natural antioxidants and variations among experimental animals. This complexity underscores the necessity for continued research in this field. We extend our gratitude to all the contributors for their innovative research. We hope that this Special Issue will inspire further scientific exploration into the effects of natural antioxidants on the health of aquatic animals, advancing our knowledge in this field.
